# The cases with opportunistic infections among HIV patients in Indonesia 2011

**DOI:** 10.1186/1471-2334-14-S2-P45

**Published:** 2014-05-23

**Authors:** Roselinda Rusli, Vivi Setiawaty

**Affiliations:** 1 Center for Biomedical and Basic Technology of Health, NIHRD, Jakarta, Indonesia

## Background

Human Immunodeficiency Virus (HIV) reduces the ability of person’s immune system to combat infection from microorganism such as Tuberculosis, Herpes simplex, Candida, Toxoplasma. Some of these microorganism are normal flora and usually do not generate a serious disease in healthy people, therefore these infections are known as opportunistic infections (OIs). The OI by tuberculosis is the most common cause of death for people infected with HIV worldwide. Since the OIs are not well recorded in Indonesia, in 2011 we conducted study in seven provinces to identify the incidence of OIs among HIV patients.

## Method

The cross sectional study was conducted in several hospitals that has voluntary counseling test (VCT) clinic. The respondents were HIV patients who visited VCT clinics in seven provinces (North Sumatera, West Sumatera, Riau Islands, South Sulawesi, North Sulawesi, Maluku, and Papua) by purposive sampling in 2011. There were 490 respondents included in this study. The statistical analyses were completed using STATA 9.0 version.

## Results

From the study we found several OI from HIV patients. 379 out of 490 respondents reported OIs. Among OIs patients, Tuberculosis, Candidiasis and Diarrhea were the most common OIs with Tuberculosis as the leading cause of OIs. There were 238 out of 490 (48.6%) infected by Tuberculosis, 202 out of 490 (41.2%) with Candidiasis and 98 out of 490 (20%) with diarrhea. Moreover, we also found Dermatitis, Anemia, Lymphadenopathy, Hepatitis C, Herpes Zoster, Toxoplasmosis, Kaposi’s sarcoma, Steven-Johnson Syndrome, Morbili, Cirrhosis Hepatis, Rheumatic Lupus and Cryptococcus Meningitis as OIs. We found highest number of Tuberculosis as the OI in Papua with 50 out of 70 (71.4%) respondents. There were 201 out of 379 (53%) cases developed more than one OI. Tuberculosis was reported mostly from patients with advanced stages of HIV.

## Conclusion

In Indonesia, Tuberculosis was the leading cause of OIs and it was developed in advance stage of HIV infection.

